# Altered Umbilical Cord Blood Nutrient Levels, Placental Cell Turnover and Transporter Expression in Human Term Pregnancies Conceived by Intracytoplasmic Sperm Injection (ICSI)

**DOI:** 10.3390/nu13082587

**Published:** 2021-07-28

**Authors:** Enrrico Bloise, Jair R. S. Braga, Cherley B. V. Andrade, Guinever E. Imperio, Lilian M. Martinelli, Roberto A. Antunes, Karina R. Silva, Cristiana B. Nunes, Luigi Cobellis, Flavia F. Bloise, Stephen G. Matthews, Kristin L. Connor, Tania M. Ortiga-Carvalho

**Affiliations:** 1 Departamento de Morfologia, Universidade Federal de Minas Gerais, Belo Horizonte, MG 31270-910, Brazil; lilianmassara@gmail.com; 2 Laboratório de Endocrinologia Translacional, Instituto de Biofísica Carlos Chagas Filho, Universidade Federal do Rio de Janeiro, Rio de Janeiro, RJ 21941-902, Brazil; jairbraga@me.ufrj.br (J.R.S.B.); cherley@biof.ufrj.br (C.B.V.A.); guinever.imperio@mail.utoronto.ca (G.E.I.); robertoantunes@fertipraxis.com.br (R.A.A.); flaviabloise@biof.ufrj.br (F.F.B.); taniaort@biof.ufrj.br (T.M.O.-C.); 3 Maternidade Escola, Universidade Federal do Rio de Janeiro, Rio de Janeiro, RJ 22240-000, Brazil; 4 Department of Physiology, Temerty Faculty of Medicine, University of Toronto, Toronto, ON M5S 1A8, Canada; Stephen.Matthews@utoronto.ca; 5 Lunenfeld-Tanenbaum Research Institute, Sinai Health System, Toronto, ON M5G 1X5, Canada; 6 Fertipraxis-Centro de Reprodução Humana, Rio de Janeiro, RJ 22640-902, Brazil; 7 Laboratório de Endocrinologia Molecular, Instituto de Biofísica Carlos Chagas Filho, Universidade Federal do Rio de Janeiro, Rio de Janeiro, RJ 21941-902, Brazil; ribeiro.ks@gmail.com; 8 Departamento de Anatomia Patológica e Medicina Legal, Universidade Federal de Minas Gerais, Belo Horizonte, MG 30130-100, Brazil; cristianabnunes@gmail.com; 9 Dipartimento della Donna, del Bambino e di Chirurgia Generale e Specialistica, Università degli Studi della Campania “Luigi Vanvitelli”, 80138 Napoli, Italy; luigi.cobellis@unicampania.it; 10 Department of Obstetrics and Gynaecology, Temerty Faculty of Medicine, University of Toronto, Toronto, ON M5S 1A8, Canada; 11 Department of Medicine, Temerty Faculty of Medicine, University of Toronto, Toronto, ON M5S 3H2, Canada; 12 Health Sciences, Carleton University, Ottawa, ON K1S 5B6, Canada; kristin.connor@carleton.ca

**Keywords:** assisted reproductive technology (ART), intracytoplasmic sperm injection (ICSI), amino acids, citrulline, glucose, lipids, free fatty acids (FFA), placenta, proliferation, apoptosis, GLUT1, SNAT2, P-gp and BCRP

## Abstract

Assisted reproductive technologies (ART) may increase risk for abnormal placental development, preterm delivery and low birthweight. We investigated placental morphology, transporter expression and paired maternal/umbilical fasting blood nutrient levels in human term pregnancies conceived naturally (*n* = 10) or by intracytoplasmic sperm injection (ICSI; *n* = 11). Maternal and umbilical vein blood from singleton term (>37 weeks) C-section pregnancies were assessed for levels of free amino acids, glucose, free fatty acids (FFA), cholesterol, high density lipoprotein (HDL), low density lipoprotein (LDL), very low-density lipoprotein (VLDL) and triglycerides. We quantified placental expression of GLUT1 (glucose), SNAT2 (amino acids), P-glycoprotein (P-gp) and breast cancer resistance protein (BCRP) (drug) transporters, and placental morphology and pathology. Following ICSI, placental SNAT2 protein expression was downregulated and umbilical cord blood levels of citrulline were increased, while FFA levels were decreased at term (*p* < 0.05). Placental proliferation and apoptotic rates were increased in ICSI placentae (*p* < 0.05). No changes in maternal blood nutrient levels, placental GLUT1, P-gp and BCRP expression, or placental histopathology were observed. In term pregnancies, ICSI impairs placental SNAT2 transporter expression and cell turnover, and alters umbilical vein levels of specific nutrients without changing placental morphology. These may represent mechanisms through which ICSI impacts pregnancy outcomes and programs disease risk trajectories in offspring across the life course.

## 1. Introduction

The advance of assisted reproductive technology (ART) allows the birth of thousands of children every year for individuals otherwise unable to conceive naturally [1,2]. However, several reports indicate that ART pregnancies may potentially be at higher risk for preterm birth, placenta praevia, placenta accreta, retained placenta, villous edema, abnormal placental growth, altered placental weight and having babies with low birthweight [3,4]. The mechanisms by which ART affect pregnancy outcomes may originate from maternal ovarian stimulation procedures; gamete/embryo manipulation and in vitro culture conditions, such as culture medium composition, culture substrate stiffness/rigidity, oxygen tension, pH, embryo culture duration; and embryo transfer methodologies, among others. In response to these stressors the embryo may adapt its growth kinetics, leading to altered fetoplacental growth and subsequent phenotypes [2].

Ultrastructural assessment of the human placenta following conception with ART revealed a thicker placental barrier and fewer microvilli at the apical membrane of syncytiotrophoblasts [5], suggesting compromised placental transport function. Placental transport efficiency may indirectly be assessed by calculating the fetal:placental (F:P) weight ratio [6]. Reduced F:P weight ratio has been observed in animal models and human population-based studies with in vitro fertilization (IVF) or intracytoplasmic sperm injection (ICSI) techniques [2,7,8,9,10]. An altered F:P ratio may be a marker of a suboptimal intrauterine environment, which is known to increase risk for cardiovascular and metabolic diseases in offspring during adult life [11]. 

The placenta supplies nutrients to the fetus by simple diffusion, facilitated or active transporter-mediated processes and by endocytosis/exocytosis [12,13]. Transplacental glucose transport is predominantly mediated by GLUT1 and GLUT3 transporters [14,15,16], whereas neutral amino acids are transported into the fetal circulation via the sodium-dependent neutral amino acid transporters (SNATs), isoforms I, II and IV [16,17]. Glucose and amino acid transport efficiency depend upon placental size and F:P weight ratio [7,18]. Term newborns from mouse pregnancies conceived by IVF have lower birthweight but exhibit higher placental weight and reduced F:P weight ratio. Additionally, mouse placentae following IVF are less efficient in transporting amino acids [7], and exhibit decreased mRNA expression of *Glut* and *Snat* transporters [7,10]. Further, impaired nutrient delivery to the fetus may contribute to the low birthweight commonly reported in ART newborns [2], and is likely to program postnatal developmental trajectories and life-long disease risk. However, less is known about the effects of specific ARTs, such as ICSI, on placental development and nutrient transport efficiency in human pregnancy, including whether changes in placental development and function influence fetal nutrient levels. 

Placental efflux transport has also been shown to vary according to placental size. Activity of the transporter P-glycoprotein (P-gp) was inversely correlated with placental size in mouse pregnancy, since larger placentae within a litter were more efficient in effluxing P-gp substrates compared to smaller placentae [19]. P-gp belongs to the ATP-binding cassette (ABC) superfamily of efflux transporters and, with the efflux transporter breast cancer resistance protein (BCRP), comprise the best investigated of the ABC transporters in the placenta, namely the multidrug resistance (MDR) transporters. MDR transporters are localized to the apical membrane of the syncytiotrophoblast and prevent the entry of a number of drugs and toxicants from the maternal circulation into the fetal compartment, therefore functioning as major placental efflux gatekeepers against fetal exposure to potentially teratogenic compounds [20]. Despite their key roles in fetal protection, placental P-gp and BCRP have not been previously investigated in the context of ICSI. In this context, since placental size may vary after ART [2,4,9], it is possible that placental expression of P-gp and BCRP in ICSI pregnancies may be impacted. 

Given the evidence from animal models and human studies showing differences in placental size and F:P weight ratio in pregnancies conceived by ART [9], and the importance of both nutrient and efflux transporters in supporting optimal fetal development and protection during pregnancy, we hypothesized that ICSI placentae would have reduced nutrient transport efficiency, and that ICSI may result in increased risk of fetal exposure to drugs and toxins in utero via altered placental MDR transport. 

## 2. Materials and Methods

### 2.1. Patients

Two groups of healthy patients (control naturally conceived (CON)) and ICSI) were recruited by the medical staff of the “Maternidade Escola” from the Universidade Federal do Rio de Janeiro—Hospital system, in Rio de Janeiro city, Brazil. The study was approved by the Research Ethics Board from “Maternidade Escola”—Universidade Federal do Rio de Janeiro (project number CAAE: 30214214.0.0000.5275). In Brazil, the ICSI procedure prevails over IVF regardless of the male infertility factor, therefore we recruited ICSI rather than IVF conceived pregnancies in our study. Inclusion criteria comprised singleton pregnancies naturally conceived (>37 weeks’ gestation; CON, *n* = 10) or conceived by ICSI with fresh embryo transfer (>37 weeks’ gestation; ICSI, *n* = 11; *n* = 9 primary infertility and *n* = 2 tubal factor infertility), delivered by elective C-section without signs of labor, recruited from June 2015 until December 2017. Informed written consent was obtained from each patient prior to inclusion in the study. Clinical data were collected during patient enrollment and interviews. Exclusion criteria comprised vaginal delivery, spontaneous onset of term labor, smoking, congenital uterine anomalies, cervical incompetence, uterine malformations, polyhydramnios, multiple gestations and fetal–maternal complications (infectious diseases, thyroid disease, asthma, cardiovascular diseases, diabetes, hypertension, preeclampsia, abruption placentae, and fetal malformation). There were no other complications or comorbidities documented in the cohort at the time of recruitment. 

### 2.2. Blood Sampling and Placental Tissue Collection

Paired maternal/umbilical fasting blood (8 h prior to C-section) and placental tissues were collected from women undergoing elective C-sections (with no labor). Maternal blood was collected from a peripheral vein immediately before the C-section procedure. Umbilical cord venous blood sampling was performed by an experienced OB-GYN, under sterile conditions, in a single clamped segment on the neonatal side of the umbilical cord immediately after birth, following neonatal separation from the cord and prior to placental delivery. Blood samples were harvested in heparinized sterile vacuum blood collection tubes and centrifuged at 3000 rpm for 15 min at 4 °C. The harvested plasma was then aliquoted and stored at −80 °C until analysis.

Placental villous tissue collection was performed as previously described [21]. In brief, placental core sampling was performed using cuts made to a core depth to avoid the maternal decidua, the chorionic plate or any area of thrombosis, infarcts or other abnormalities. Therefore, only placental villous tissue from term placentae was selected. Placental fragments were washed in 0.9% sterile saline, quickly dried and immediately placed in RNALater (Thermo Fischer Scientific, São Paulo, Brazil) for downstream mRNA expression analysis, or fixed overnight in buffered 4% paraformaldehyde (Sigma-Aldrich, São Paulo, Brazil), for histopathological, TUNEL and immunohistochemistry analyses.

### 2.3. Analysis of Maternal and Umbilical Venous Blood Nutrient Contents 

Assessment of nutrient concentrations in the umbilical venous blood and matched maternal blood was performed according to routine protocols by a commercial laboratory specialized in conducting genetic, biochemical, and metabolic analyses of neonates, (DLE^®^ Genética Humana e Doenças Raras, located in Rio de Janeiro, Brazil). 

Free amino acid levels of aspartic acid, glutamic acid, alanine, arginine, phenylalanine, glycine, methionine, ornithine, serine, tyrosine, threonine, tryptophan, valine, asparagine, isoleucine, leucine, lysine, taurine, citrulline and histidine, were measured by Liquid Chromatography/Tandem Mass Spectrometry (LC-MS/MS) method. Serum glucose, cholesterol, HDL, LDL, VLDL and triglyceride levels were measured by the enzymatic colorimetric method. Free fatty acids (FFA) were quantified by the kinetic spectrophotometry method, according to the DLE laboratory specifications for each test. 

### 2.4. Quantitative Real-Time PCR (qPCR)

qPCR was performed as described previously [21,22,23]. Total RNA was isolated from placental villous tissue (~30 mg) using TRIzol Reagent (TRIzol Reagent; Life Technologies, USA). RNA concentration and purity were assessed with Implen NanoPhotometer (Implen GmbH, Munich, DE, Germany). Only samples with a 260 nm/280 nm absorbance ratio higher than 1.8 were included in the study. For cDNA synthesis, 1000 ng of total RNA were reverse-transcribed into cDNA using a High Capacity cDNA Reverse Transcription Kit (Applied Biosystems, San Francisco, CA, USA). mRNA levels of genes of interest were assessed with intron-spanning primers (Table 1) by qPCR using HOT FIREPol Evagreen qPCR Supermix (Solis, Denmark) and the Master Cycler Realplex system (Eppendorf, Germany). The following cycling conditions were performed: combined initial denaturation at 50 °C (2 min) and 95 °C (10 min), followed by 40 cycles of denaturation at 95 °C (15 s), annealing at 60 °C (30 s) and extension at 72 °C (45 s). Assay efficiency ranged from 95–105%. Genomic DNA contamination was ruled out using reverse transcriptase-negative samples and melting curve analysis extracted from each reaction. Gene expression was normalized to the geometric mean of three stably expressed reference genes: peptidyl-propyl isomerase B (*PPIB)*, 14-3-3 protein zeta/delta (*YWHAZ*) and TATA-binding protein (*TBP*) (Table 1). Relative gene expression was calculated according to LinReg method, qPCR efficiency was 100 ± 10%. 

### 2.5. Histopathological, Immunohistochemistry and TUNEL Assessment of CON and ICSI Placentae

Paraffin-embedded placental tissue was cut into 5-μm stepped serial cross-sections, and every sixth section was stained with either hematoxylin and eosin (H&E) for histopathological analysis or subjected to immunohistochemical or TUNEL analysis. 

Morphometric analysis consisted of evaluating the volumetric proportions of the cellular components of the placentae as well as the presence of trophoblast pathologies that could be attributed to the method of conception. The volumetric proportion analysis in CON and ICSI placentae was undertaken by two separate examiners blinded to groups estimating the following histological components: syncytiotrophoblasts, syncytial knots, cytotrophoblasts, connective tissue, and blood vessel. Captured images were superimposed with a grid of equidistant points (25 μm). 1000 points were counted per placenta; equivalent to an area of, on average, 655,288.7 μm^2^. The volumetric ratio (VR) of each component was calculated as VR = NP × 100/1000, where NP = number of equivalent points for each histological component [22,23]. Analysis and image acquisition were performed in a Zeiss Axiolab 1 photomicroscope (Carl Zeiss, White Plains, NY, EUA), coupled to a CCD camera (Leica DFC345FX). Morphometric analysis was performed using the Fiji ImageJ 1.0 (ImageJ, Madison, WI, USA) program.

Histopathological analysis was undertaken by a single experienced pathologist blinded to groups, in three random placental sections per patient. Placental pathological features investigated were: villous edema, microcalcification, chronic villitis (presence of inflammatory cells infiltrating the villous stroma), ischemic or infarction changes (defined by the presence of localized dead or devitalized chorionic villi and noticeable villous agglutination or early infarction), avascular villi, increased peri-villous fibrin (accumulation of fibrin or fibrinoid material in the intervillous space), and intervillous thrombi (laminated clots within the intervillous space) [29,30,31]. These pathological features were deemed either present or absent in CON and ICSI placentae [30]. 

For immunohistochemistry, following deparaffinization in three xylene immersions and rehydration in descending gradients of ethanol, endogenous peroxidase activity was blocked with 3% H_2_O_2_ in methanol for 10 min and washed in PBS. Antigen retrieval was performed by preheating sections in 10 mmol/L sodium citrate (pH 6.0). Sections were again washed in PBS before blocking in 3% PBS/BSA for 1 h. Sections were incubated overnight at 40 °C, with the following primary antibodies: anti-Ki67 antibody (Spring Bioscience, Pleasanton, CA, USA), anti-GLUT1 (1: 100—ab652, Abcam, SP, Brasil), anti-SNAT 2 (1: 200—LS-C179270, LSBio, Seattle, WA, EUA), anti-P-gp (1:500—Mdr1[sc-55510]; Santa Cruz Biotechnology, Dallas, TX, USA) and anti-Bcrp (1:100—Bcrp [MAB4146]; Merck Millipore, Burlington, MA, USA). Followed by slide washing and incubation with biotin-conjugated secondary antibody (SPD-060-Spring Bioscience, Pleasanton, CA, USA) for 1 h. Sections were then re-washed in PBS (3× for 10 min each time), incubated with streptavidin-HRP (30 min; SPD-060-Spring Bioscience, Pleasanton, CA, USA) and submitted to chromogenic detection of horseradish peroxidase (HRP) activity by 3,3′-diaminobenzidine (DAB) reagent (DAB peroxidase substrate kit, SPD-060-Spring Bioscience, Pleasanton, CA, USA). The immune reaction was also performed in the absence of the primary antibody (negative control) to monitor nonspecific binding of the secondary antibody.

The terminal deoxynucleotidyl transferase dUTP nick end labeling (TUNEL) analysis was undertaken to assess apoptotic nuclei ratio, using the ApopTag^®^ In Situ Peroxidase Detection Kit (S7100—Merck Millipore, Danvers, MA, USA), according to the manufacturer’s instructions. TUNEL reaction was also performed omitting TdT as negative control. Slides were counterstained with hematoxylin, dehydrated in ascending grades of ethanol, clarified in three xylene immersions and cover-slipped. Slides were visualized with a high-resolution Olympus DP72 (Olympus Corporation, Tokyo, Japan) camera coupled to the Olympus BX53 light microscope (Olympus Corporation, Tokyo, Japan). Scoring of immunosignals was performed as previously described with adaptations [32,33,34], using STEPanizer software [35]. A total of 30 digital images were captured per placental fragment of each patient. In each image, Ki-67 and TUNEL nuclei immunostaining were quantified and the resulting value was divided by the area of the total image, which yielded an estimate for the number of proliferative or apoptotic nuclei present in the whole tissue (number of nuclei/area of tissue). Quantification of GLUT 1, SNAT2, P-gp and BCRP immunostaining was performed using the Image Pro Plus 5.0 software (Media Cybernetics, Maryland, Rockville, MD, USA) mask tool, where immunostaining of the percentage area of viable tissue was quantified (with negative/empty spaces excluded) [32,33,34]. 

### 2.6. Statistical Analysis

Outliers were identified using the ROUT method (GraphPad Prism 7.0 software). Differences between conception groups for clinical data, immunohistochemistry, qPCR and morphological comparisons were determined by normality test using D’Agostino-Pearson normality test followed by unpaired *t*-test or non-parametric Mann-Whitney test. Data are presented as means ± standard deviation (SD) or median and interquartile range (IQR). Nutrient outcome measures in maternal and cord blood were tested for normality and unequal variances (Levene test, JMP v14.3). Data that were non-normal were transformed to achieve normality, where possible. Differences between conception groups for outcome measures were determined by *t*-test, or Welch ANOVA, or Wilcoxon test for non-parametric data (*p* < 0.05, JMP v14.3). Nutrient data are presented as means ± SD or median and IQR. Data transformed for analyses are presented as untransformed values. There were no sex differences in any nutrients investigated in the cord blood (data not shown), thus analyses between CON and ICSI groups are inclusive of newborn sex. There was a total of 10 CON and 11 ICSI participants who had nutrient levels measured in maternal blood and umbilical vein blood. Some participants did not have a measured value for a nutrient, and/or statistical outliers were removed, resulting in lower final *n*-numbers for specific nutrient measures (which are reported for each nutrient in the tables). Fisher’s exact test was performed to evaluate placental histopathological findings between groups (GraphPad Prism 7.0 software). Correlations between maternal pre-pregnancy BMI and levels of nutrients in maternal circulation, or between levels of nutrients in maternal circulation and umbilical vein circulation, were determined by Pearson or Spearman correlation analyses. Data are presented as Pearson’s correlation coefficient or Spearman’s rho. Statistical differences were set at *p* < 0.05.

## 3. Results

### 3.1. Clinical Data 

There were no differences in maternal age, initial (first trimester) and final (term) body mass index (BMI), maternal weight gain across pregnancy, gestational age at delivery, birth weight, placental weight or F:P weight ratio between conception groups (Table 2).

### 3.2. ICSI Impairs Placental SNAT 2 Immunostaining

**Figure 1:**
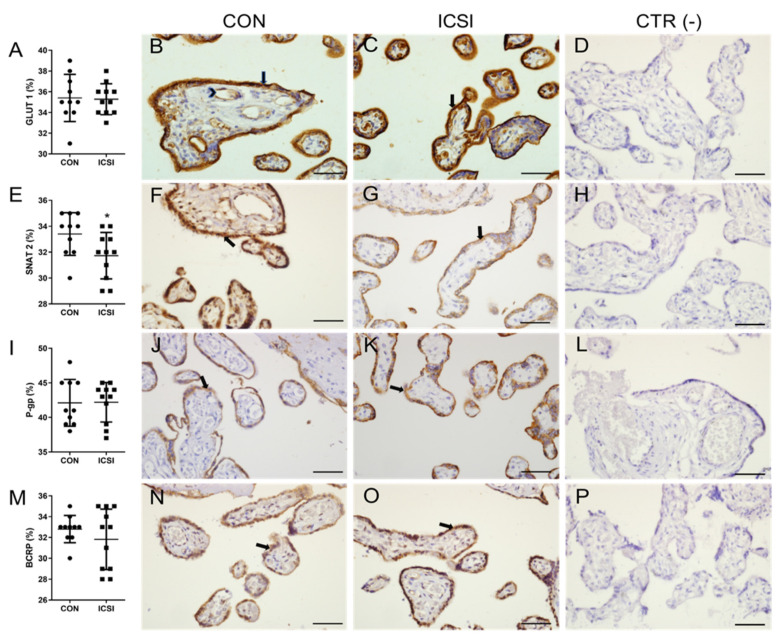
The amino acid transporter SNAT2 is downregulated in the human ICSI placenta. Semiquantitative staining scores (**A**,**E**,**I**,**M**) and corresponding representative immunostaining photomicrographies of placental GLUT1 (**B**,**C**), SNAT2 (**F**,**G**), P-gp (**J**,**K**) and BCRP (**N**,**O**) in control naturally conceived (CON; *n* = 10/group) and ICSI (*n* = 11/group) term placentae. All transporters were predominantly localized to the membrane and cytoplasm of syncytiotrophoblasts. Staining of GLUT1, SNAT2 and BCRP were also detected, to a lesser extent, in endothelial cells of fetal blood vessels and in the cytoplasm of placental connective tissue. (**D**,**H**,**L**,**P**) negative controls. Data are presented as media ± SD. (**A**,**E**,**I**,**M**) statistical differences were analyzed using Unpaired *t*-test. * *p* < 0.05. Arrows indicate the syncytium; arrow heads indicate fetal blood cells. Scale bar = 50 μm.

We determined the placental mRNA expression of major transporter systems. We did not detect statistical differences in the mRNA expression levels of the neutral amino acid transporters, *SLC38A1* (encoding SNAT1), *SLC38A2* (SNAT2) and *SLC38A4* (SNAT 4); in the glucose transporters, *SLC2A1* (GLUT1) and *SLC2A3* (GLUT3); and in the MDR transporters *ABCB1* (P-gp) and *ABCG2* (BCRP) (Supplementary Figure S1). GLUT1 and SNAT2 are two major placental glucose and amino acid transporters, and their immunostaining was detected in the membrane and cytoplasm of syncytiotrophoblasts, in endothelial cells of fetal blood vessels and in the cytoplasm of placental connective tissue (to a lesser extent). SNAT2 immunostaining was decreased in ICSI placentae (*p* < 0.05) and there were no changes in the semiquantitative expression levels of placental GLUT1 transporter (Figure 1A–H). P-gp and BCRP were immunolocalized in the apical membrane and cytoplasm of synctiotrophoblasts, whereas BCRP was also localized in endothelial cells of fetal blood vessels and in placental connective tissue. There were no changes in immunostaining of P-gp and BCRP between groups (Figure 1I–P). 

### 3.3. Venous umbilical Cord Blood from ICSI Term Pregnancies Exhibit Specific Changes in Nutrient Levels

**Figure 2:**
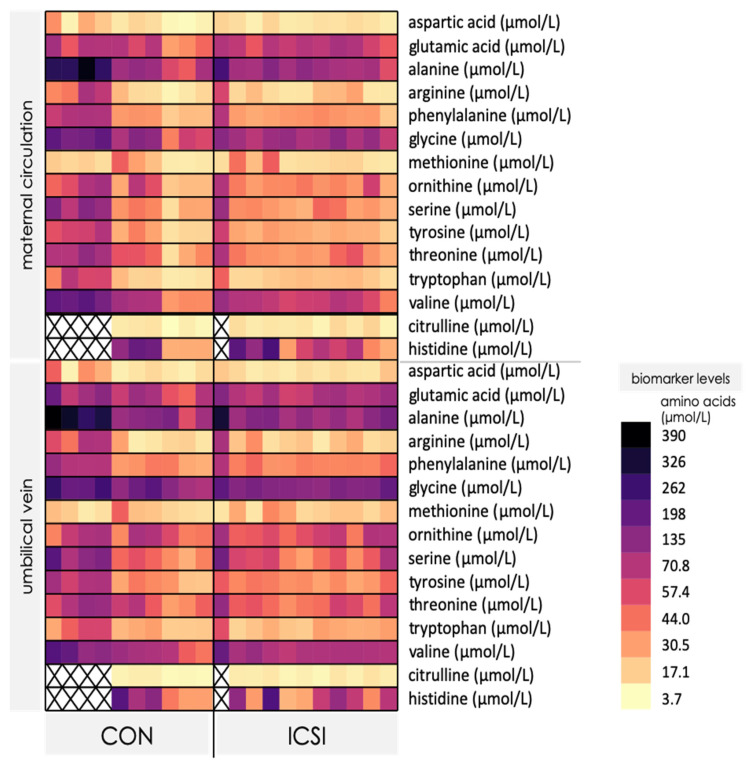
Heatmap of free amino acid levels in maternal circulation and umbilical vein in control, naturally conceived (CON; *n* = 10) and ICSI (*n* = 11) term pregnancies. Each column represents the nutrient profile of an individual pregnancy (maternal and umbilical vein). Darker colors indicate higher biomarker concentrations whereas lighter colors indicate lower biomarker concentrations. X = no value measured. Refer to Supplementary Tables S1 and S2 for values.

We next evaluated maternal and umbilical cord blood levels of free amino acids in CON and ICSI pregnancies to identify possible changes that could be attributed to the method of conception. Levels of aspartic acid, glutamic acid, alanine, arginine, phenylalanine, glycine, methionine, ornithine, serine, tyrosine, threonine, tryptophan and valine were consistently detected in the maternal and venous umbilical cord blood in CON and ICSI pregnancies, whereas citrulline and histidine were not detected in some participants (Figure 2). 

Levels of asparagine, isoleucine, leucine, lysine and taurine, were not detected in most participants and were therefore not analyzed for statistical differences (Supplementary Tables S1 and S2). Overall, there were more CON mothers with blood levels of free amino acids within the reference ranges than ICSI mothers, despite that the majority of the subjects had amino acid levels below the reference ranges (Supplementary Figure S2). There were no differences in the levels of free amino acids in maternal circulation between CON and ICSI (Table 3 and Supplementary Table S1), however, citrulline concentration was increased in venous cord blood of ICSI-conceived newborns compared to newborns conceived naturally (*p* < 0.05, Table 3). Umbilical cord levels of the other amino acids measured (aspartic acid, glutamic acid, alanine, arginine, phenylalanine, glycine, methionine, ornithine, serine, tyrosine, threonine, tryptophan, valine and histidine) did not differ between CON and ICSI (Supplementary Table S2).

**Figure 3:**
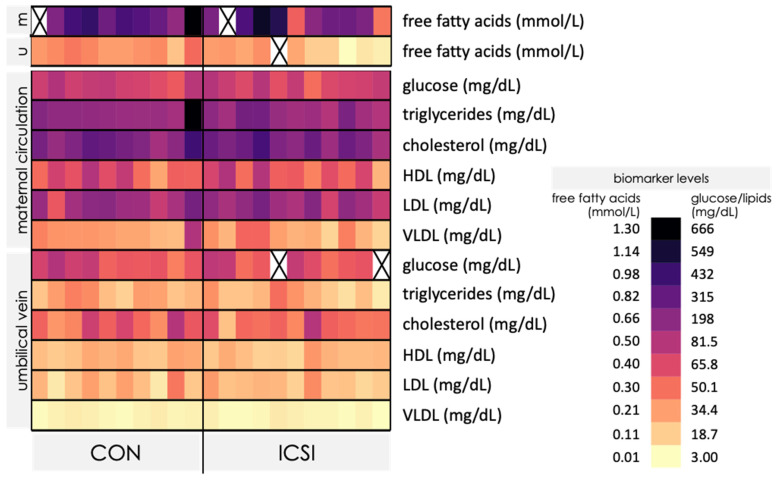
Heatmap of free fatty acids, glucose and lipid biomarker levels in maternal circulation and umbilical vein in control naturally conceived (CON; *n* = 10) and ICSI (*n* = 11). Each column represents the nutrient profile of an individual pregnancy (maternal and umbilical vein). Darker colors indicate higher biomarker concentrations and lighter colors indicate lower biomarker concentrations. X = no value measured. Refer to supplementary Tables S1 and S2 for values. m = maternal circulation. u = umbilical vein.

We observed higher maternal blood levels of FFA, lipids and lipoproteins in the maternal circulation compared to umbilical cord blood in CON and ICSI pregnancies (Figure 3). Overall, both CON and ICSI mothers demonstrated normal blood levels of HDL cholesterol, but both groups overall exhibited high levels of all other lipids/lipoproteins (Supplementary Figure S3). There were no differences in the levels of FFA (Table 3), glucose, lipids or lipoproteins in maternal blood between CON and ICSI groups (Supplementary Table S1). However, FFA levels were lower in cord blood of ICSI-conceived newborns compared to newborns conceived naturally (*p* < 0.01, Table 3), whereas levels of other lipids/lipoproteins and glucose did not differ between groups (Supplementary Table S2).

Next, we explored relationships between key clinical variables and outcome measures in our cohort. Maternal pre-pregnancy BMI was not correlated with circulating maternal nutrient levels, except a slight negative correlation between pre-pregnancy BMI and glutamic acid levels (r = −0.439, *p* = 0.046). Second, we observed significant positive correlations between all circulating nutrient levels in mothers with their levels in the umbilical vein, with the exception of triglycerides, HDL, VLDL and methionine (Table 4). 

We also explored potential relationships between umbilical vein FFA and citrulline levels. We did not find any relationship between levels of these nutrients (r = −0.362, *p* = 0.18) for the whole cohort, or when exploring relationships within each of the CON (*p* = −0.529, *p* = 0.28) and ICSI groups (*p* = 0.185, *p* = 0.63).

### 3.4. Placental Proliferation and Apoptosis Are Increased in the Human ICSI Placenta

**Figure 4:**
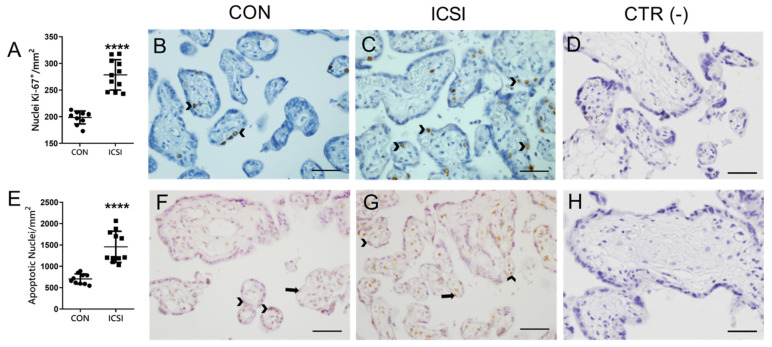
Placental proliferation and apoptosis are increased in the human ICSI placenta. Semiquantitative staining scores (**A**,**E**) and corresponding representative immunostaining photomicrographies of Ki67 (**B**,**C**) and TUNEL (**F**,**G**) obtained from term human naturally (CON; *n* = 10) conceived and ICSI (*n* = 11) placentae. (**D**,**H**) negative controls. (**A**,**E**) data are presented as mean ± SD. Statistical differences were analyzed using Unpaired *t*-test. **** *p* < 0.0001. Arrows indicate syncytium nuclear staining; arrowheads indicate cytotrophoblastic nuclear staining. Scale bar = 50 μm.

**Figure 5:**
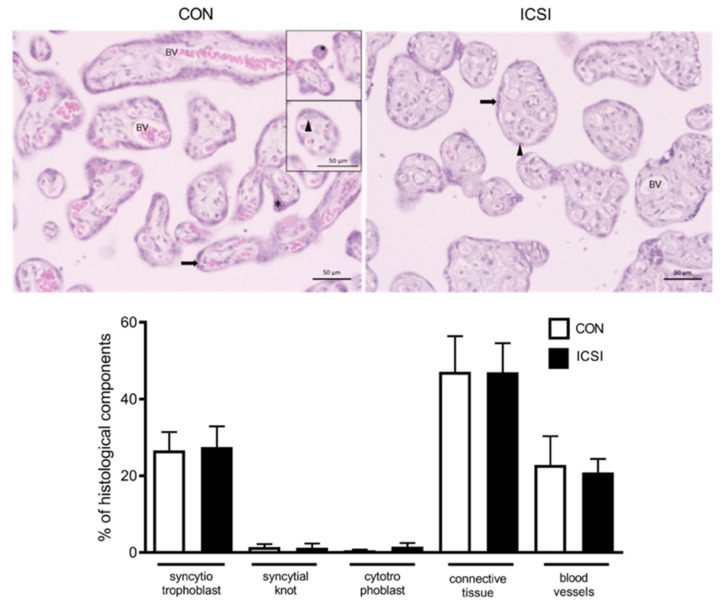
ICSI does not alter placental volumetric proportion. Representative histological photo-micrographies of control naturally conceived (CON; *n* = 10) and ICSI (*n* = 11) placentae and correspondent volumetric proportion analyses of the number of syncytiotrophoblasts, cytotrophoblasts, connective tissue, blood vessels, and syncytial knots. Data presented as mean ± SD. *p* < 0.05. Arrows indicate the syncytium, arrow heads indicate cytotrophoblasts, indicate the syncytium knots and BV= blood vessels (fetal). Scale bar = 50 μm.

Since altered proliferation and apoptosis have been previously described in the mouse IVF placenta [7,36], we investigated whether the human ICSI placenta would exhibit differences in parameters related to cellular proliferation (placental staining of the Ki67 proliferation marker) and apoptosis (TUNEL staining). Increased Ki67 staining (*p* < 0.0001) was detected in the nuclei of cytotrophoblasts and connective tissue (to a lesser extent), whereas increased apoptotic immunosignals (*p* < 0.0001) were visible in the nuclei of syncytiotrophoblasts, cytotrophoblasts and connective tissue of ICSI placentae (Figure 4). Next, to evaluate whether these changes would be associated with any specific placental pathologies, we undertook histopathological analyses. We did not detect any placental pathologies that could be specifically attributed to the method of conception (Supplementary Table S3). Further, we also investigated the impact of ICSI in the number of placental histological components by performing a volumetric proportion analyses. No changes in the number of syncytiotrophoblasts, cytotrophoblasts, connective tissue, blood vessels or syncytial knots were observed between groups (Figure 5).

## 4. Discussion

This is the first study to comprehensively quantify placental nutrient and MDR transporters and maternal and umbilical nutrient levels, in ICSI compared to naturally conceived pregnancies, to better understand the mechanisms that may explain poor pregnancy outcomes in pregnancies conceived with ICSI. Using a cohort from Brazil, we found that ICSI pregnancies had placentae with decreased SNAT2 protein expression, had significant increase in venous umbilical cord citrulline and decreased FFA levels at term. We also observed increased markers of proliferation and apoptosis in ICSI placentae. These alterations may potentially disrupt placental development and transport potential.

In our study, contrary to our hypothesis, there were no changes in placental or birth weights or F:P weight ratio, suggesting that alterations in these parameters may not be common findings in pregnancies conceived by ICSI (vs. other forms of ART), or, alternatively, that other comorbidities often associated with sub- and infertility, but controlled for or absent in our study (e.g., maternal complications, elevated maternal BMI), explain more of the differences seen in fetoplacental weights following ART procedures [2,37]. This is consistent with the lack of differences between ICSI and CON participants in our study for maternal age, weight gain and BMI, amongst other clinical data. Further, although greater placental weight and lower F:P ratio were reported in pregnancies conceived by ART (ICSI and IVF) in a large Scandinavian based-population study [9], these differences in findings may also be attributed to sample size and/or to population ethnicity and other demographics (Scandinavian vs. Brazilian). Our findings are also in contrast with those described in mice conceived by IVF, which exhibited lower fetal weight, higher placental weight and lower F:P ratio [7,10]. It is important to note that there have been reports showing species-specific placental and fetal phenotypic adaptations in response to ART [2]. 

We detected lower protein expression levels of the neutral amino acid transporter SNAT2 in ICSI placentae, whereas GLUT1 (glucose) and P-gp/BCRP (drug) immunostaining remained unchanged. mRNA levels of *SLC38A2* (encoding SNAT2) and other transporter systems were not different between ICSI and CON groups, suggesting that ICSI alters placental protein levels of SNAT2 without changing its corresponding mRNA levels, at least in term no labor C-section placentae. Placental SNAT2 has been reported to be highly regulated by a variety of environmental factors including in vitro culture conditions [7], hormones (glucocorticoid exposure) [24,38,39], pH, oxygen tension [40], high fat diet [41], maternal protein restriction [42] and in obstetric conditions such as intrauterine growth restriction (IUGR) [43,44]. SNAT2 transports neutral amino acids such as alanine, asparagine, cysteine, glutamine, glycine, histidine, methionine, proline and serine and its activity is upregulated when cell growth and increased protein synthesis is required [17]. This is consistent with our finding of increased rates of cell proliferation and apoptosis in ICSI placentae, which may explain, at least in part, the altered placental SNAT2 expression levels observed. 

Despite altered SNAT2 protein levels, there were no changes in SNAT2 amino acid substrates in the venous umbilical cord blood of ICSI pregnancies. However, we did find higher cord blood citrulline levels with ICSI. Citrulline was detected in 6 out of 10 CON pregnancies and 9 out of 11 ICSI pregnancies. This is the first report to show alterations in citrulline levels that could be attributed to ICSI procedures. Citrulline can originate from ornithine by actions of the ornithine carbamyl/carbamoyl transferase (OTC) or be released as by-product resulted from enzymatic nitric oxide synthesis from arginine by endothelial nitric oxide synthase (eNOS), inflammatory (iNOS) or neuronal NOS (nNOS) [45,46,47]. Of interest, placental citrulline synthesis and transport is not well understood, but may occur via distinct transporter systems as reported in other cell types [44,46]. Enterocytes may uptake citrulline using B (0,+), L, and b (0,+) amino acid transport systems [48], whereas in human umbilical vein cells (HUVECs), citrulline, along with arginine, is transported via the cationic amino acid transporter system y+/CATs [49]. Citrulline transmembrane transport occurring via Na+-dependent systems in rat small intestine and proximal tubular kidney cells have also been documented [47,50,51]. Of importance, citrulline levels in fetal plasma were increased in nutrient restricted (70% of control diet) pregnant baboons at mid-pregnancy, with no alterations in fetal and placental weight, i.e., prior to the onset of IUGR [45]. Importantly, nitric oxide (NO) derived from (eNOS) activity controls placental vascular tone and an imbalance of endothelial eNOS/arginase activity may contribute to vascular dysfunction in IUGR umbilical and placental vessels [52]. In our study, we excluded pregnancies with complications such as IUGR and pre-eclampsia, but given that ART-conceived pregnancies have higher IUGR and pre-eclampsia risk [4], further studies should investigate the maternal and fetal citrulline levels in ICSI pregnancies complicated by these conditions. 

We observed a decrease in serum FFA levels in venous cord blood of ICSI newborns. Placental transport of FFA occurs via several transport mechanisms [16,53] and when visually comparing the heatmaps of the maternal versus the umbilical vein lipids and lipoproteins, it is possible to identify lower levels of these lipid biomarkers in the umbilical vein, showing that lipid transfer into the fetal circulation is largely controlled by the placenta. Future studies are necessary to investigate placental lipid metabolism and transfer in ICSI pregnancies to understand the mechanisms leading to lower FFA levels in ICSI newborns, and the impact this may have on fetal development. Although there were no differences in maternal levels of lipids and lipoproteins between ICSI and CON, overall, both groups of mothers had levels of FFA, cholesterol, triglycerides and lipoproteins above the recommended reference ranges. This may suggest that the placenta is adapting to lipid excess in order to regulate supply to the fetus. Further, taken with our finding that most mothers had lower circulating levels of free amino acids than recommended reference ranges for adults, our data suggests that participants in our study had a suboptimal nutritional profile. Future studies could cross-validate blood biomarker findings with validated food frequency questionnaires to better understand participant nutritional profiles and whether these, or the ART procedure itself, contribute to the outcomes observed, including lower FFA levels in cord blood from ICSI pregnancies. Since maternal malnutrition alters materno–fetal FFA transfer in pregnancy [54], disrupts placental expression of fatty acid transporters in mice [53] and in ewes [55] and significant lower cholesterol levels in the ICSI mouse placenta have been reported [56], it is conceivable that placental fatty acid transport is altered following ICSI in human pregnancies. This clearly requires further investigation.

Correlation analyses identified that, apart from triglycerides, HDL, VLDL and methionine, umbilical vein nutrient levels were positively associated with maternal levels of these nutrients. This demonstrates that even though the syncytium trophoblast barrier controls fetal nutrient transfer through the action of diverse mechanisms [13,16], the umbilical cord nutrient profile is directly dependent upon the maternal nutritional status. In our cohort, maternal pre-pregnancy BMI had limited influence on maternal blood nutrient levels. Further, the lack of correlation between umbilical cord citrulline and FFA levels in ICSI pregnancies suggests that ICSI impacts levels of these nutrients in the umbilical cord, possibly through independent, currently unknown mechanisms. 

The placenta has high energetic demands throughout pregnancy, which increase exponentially at term [57]. Placentomegaly has been detected at term ICSI mouse [58] and human (IVF and ICSI-conceived) placentae [9], accompanied by significantly lower birthweights compared to spontaneously conceived pregnancies [9]. With this in mind, and the altered placental turnover we observed by increased rates of proliferation and apoptosis in ICSI placentae, it is possible that ICSI placentae are reallocating energetic and metabolic resources to a greater degree than CON, such that these metabolic substrates are used by the placenta to guarantee proper placental size (or its overgrowth)—a hypothesis that clearly requires further investigation. This, however, could have detrimental effects for the fetus, such as decreased transfer of key nutrients, consistent with our finding of lower umbilical cord serum FFA levels in ICSI newborns. 

A consequence of decreased levels of specific nutrients in the fetal blood is reprogramming of fetal metabolism which may, in the short-term, ensure proper fetal growth, (here reflected by normal birthweight found in the ICSI group), but in the long-term, this adaptation may predispose offspring to cardiometabolic diseases in adult life, as demonstrated extensively in the developmental origins of health and disease (DOHaD) field [59]. Further, it is possible that changes in nutrient levels in the umbilical cord of ICSI newborns is one of the mechanisms through which excessive gamete and embryo manipulation inherent to ART procedures may program ART conception to altered developmental trajectories and life-long disease risk.

Increased placental apoptosis may be indicative of intrauterine stress, as commonly associated with excessive manipulation of gametes and embryos in ART reproductive cycles [2]. Importantly, given the increase in placental Ki67 staining, we can infer that ICSI placentae are capable of circumventing increased apoptotic rates and maintain cellular allostasis by increasing levels of cell proliferation. The increased proliferation/cell division rates herein observed were not associated with changes in the volumetric ratio of the various placental cell types or specific placental-related pathologies, and thus, allowed adequate placental and fetal growth, at least in later stages in pregnancy. 

As to the other transporter systems investigated in this study, we did not observe differences in placental expression levels of GLUT1, P-gp and BCRP transporters in the CON and ICSI groups. Dong et al. [60] found increased levels of GLUT1 in placentae from ART pregnancies, which contrasts with our findings. Such differences may result from patient inclusion criteria in both studies, since the ART group in Dong’s study consisted of pregnancies conceived by IVF and ICSI, delivered both by vaginal and C-section modes [60], whereas in this report, we only recruited ICSI pregnancies which delivered by C-section with no labor. Accordingly, we did not find differences in glucose levels in the maternal and venous umbilical cord blood, which matches our findings in placental GLUT1 expression. Further, the lack of differences in placental P-gp and BCRP expression between groups, suggests a preserved protective function of these efflux transporters in ICSI pregnancies, at least in later stages of pregnancy.

Strengths of our study include our comprehensive quantification of the maternal and umbilical cord blood nutrient milieux in ICSI and naturally conceived pregnancies, which allowed us to better capture potential differences in nutrient availability and transfer to the fetus than inferring these based on placental transporter expression alone. Further, our study is one of the few to examine ICSI exposures or placental outcomes in pregnant populations from the Global South, filling a large gap in our understanding of the mechanisms driving fetoplacental development and the programming of later health trajectories in underrepresented populations. As our groups were similar for clinical characteristics, particularly variables associated with subfertility and adverse pregnancy outcomes, all differences described are likely to be attributed to the ICSI method of conception and/or to the intrinsic risk factors inherent to the various causes of the couples’ infertility that were not measured here. One potential limitation of our study is the relatively small number of patients enrolled in each group, warranting caution when considering the implications or translatability of our findings to other contexts. Nevertheless, our findings provide preliminary data for larger observational trials. For example, our groups were well-matched for key potential covariates including mode of delivery (all were C-section with no labor), initial and final maternal BMI and newborn fetal sex rates, and we did not include pregnancies with fetal–maternal, endocrine or metabolic complications or other comorbidities. Thus, we were able to study the effects of ICSI more accurately. In addition, due to our limited sample size, we were not able to robustly investigate the effects of sex on placental outcomes. Future studies should be powered to look at sex differences in the placenta from naturally conceived and ICSI conceived pregnancies. Another potential limitation of our study is the semiquantitative nature of the protein measurements. Nevertheless, such analyses enabled us to identify transporter protein expression not only in the syncytiotrophoblast layer of the placenta, but also in endothelial cells of fetal blood vessels and in the connective tissue. 

## 5. Conclusions

We provide some of the first evidence for altered cord blood citrulline and FFA levels in human term pregnancies conceived by ICSI, associated with increased placental cellular turnover (increased rates of placental cell proliferation and apoptosis) and reduced SNAT2 protein abundance. Our data suggest that ICSI may have subtle, but important, effects on placental function without gross alterations in placental size, histology or pathology. Newborns conceived through ICSI may thus be potentially exposed to stressors in utero, which can result in metabolic adaptations, at least in late pregnancy, highlighting the need for follow-up studies to understand postnatal endocrine and metabolic phenotypes and how ICSI and altered placental function may explain these. The present study improves our understanding of the mechanisms that may contribute to poor pregnancy and postnatal outcomes in ICSI pregnancies and offspring, and adds to the dearth of evidence on placental development and function in pregnancies from the Global South.

## Figures and Tables

**Table 1:** List of Primer sequences used in this study.

Gene	Primer Sequences	Reference
*SLC38A1* (SNAT1)	F: 5′-GTGTATGCTTTACCCACCATTGC-3′ R: 5′-GCACGTTGTCATAGAATGTCAAGT-3″	[24]
*SLC38A2* (SNAT2)	F: 5′-AGATCAGAATTGGCACAGCATA-3′ R: 5′-ACGAAACAATAAACACCACCTTAA-3′	[24]
*SLC38A4* (SNAT4)	F: 5′-GAGGACAATGGGCACAGTTAGT-3′ R: 5′-TTGCCGCCCTCTTTGGTTAC-3′	[24]
*SLC2A1* (GLUT1)	F: 5′-ATCAACCGCAACGAGGAGAAC-3′ R: 5′-CACCACAAACAGCGACACGAC-3′	[25]
*SLC2A3* (GLUT3)	F: 5′-TCAGGCTCCACCCTTTGCGGA-3′ R: 5′-TGGGGTGACCTTCTGTGTCCCC-3′	[26]
*ABCB1* (P-gp)	F: 5′-AGCAGAGGCCGCTGTTCGTT-3′ R: 5′-CCATTCCGACCTCGCGCTCC-3′	[27]
*ABCG2* (BCRP)	F: 5′-TGGAATCCAGAACAGAGCTGGGGT-3′ R: 5′-AGAGTTCCACGGCTGAAACACTGC-3′	[27]
*PPIB*	F: 5′ GAGACTTCACCAGGGG -3′ R: 5′- CTGTCTGTCTTGGTGCTCTCC-3′	[28]
*YWHAZ*	F: 5′-ACTTTTGGTACATTGTGGCTTCAA-3′ R: 5′-CCGCCAGGACAAACCAGTAT-3′	[28]
*TBP*	F: 5′-TGCACAGGAGCCAAGAGTGAA-3′ R: 5′-CACATCACAGCTCCCCACCA-3′	[28]

**Table 2:** Clinical and biometric profile of the pregnancies enrolled in the study.

Parameter	CON (*n* = 10)	ICSI (*n* = 11)	*p*-Value
Maternal age (years)	32.50 ± 7.71	35.36 ± 6.61	NS
Gestational age at delivery (days)	271.6 ± 2.37	272.0 ± 3.79	NS
Weight gain (kg; 12 weeks-birth)	10.31 ± 4.44	14.30 ± 5.90	NS
Initial maternal BMI (kg/m^2^)	25.62 ± (19.0–29.4)	23.58 ± (21.7–28.3)	NS
Final maternal BMI (kg/m^2^)	29.57 ± 3.17	28.59 ± 2.53	NS
Newborn sex	M (4); F (6)	M (6); F (5)	
Birthweight (g)	3365 ± 318	3199 ± 194	NS
Placental weight (g)	592.6 ± (359–689)	547.8 ± 22.21 (438–655)	NS
Newborn head circumference (cm)	35.00 ± 0.97	34.41 ± 1.36	NS
Newborn length (cm)	48.20 ± 1.69	48.77 ± 1.65	NS
Fetal/Placental Weight ratio	5.81 ± 0.98	5.94 ± 0.91	NS
Apgar 1′/Apgar 5′	9/10	9/10	NS

Data are mean ± SD or median (IQR). Groups were analyzed using Unpaired *t*-test or Mann-Whitney Test. newborn. CON = naturally conceived, ICSI = intracytoplasmic sperm injection. NS = not significant.

**Table 3:** Citruline and free fatty acid concentrations in maternal and umbilical vein circulations with natural conception and ICSI.

Nutrient	CON	ICSI	*p*-Value
Maternal circulation			
Citrulline (μmol/L)	8.2 ± 3.0 (6)	10.7 ± 3.1 (10)	NS
Free fatty acids (mmol/L)	0.89 ± 0.19 (9)	0.75 ± 0.28 (10)	NS
Umbilical Vein Circulation			
Citrulline (μmol/L)	6.5 ± 1.6 (6)	8.5 ± 1.9 (10)	0.0385
Free fatty acids (mmol/L)	0.24 ± 0.05 (10)	0.14 ± 0.08 (10)	0.0057

Data are mean ± SD. *n* for each variable indicated in parentheses. NS = not significant. CON = naturally conceived; ICSI = intracytoplasmic sperm injection.

**Table 4:** Correlations between maternal circulating nutrient levels and the same nutrient level in umbilical vein from CON and ICSI pregnancies.

Nutrient	Spearman’s Rho	*p*-Value
Free fatty acids (mmol/L)	0.6757	0.002
Glucose (mg/dL)	0.5271	0.02
Triglycerides (mg/dL)	0.2182	NS
Cholesterol (mg/dL)	0.4717	0.03
HDL (mg/dL)	0.0095	NS
LDL (mg/dL)	0.5436	0.01
VLDL (mg/dL)	−0.0250	NS
Aspartic acid (μmol/L)	0.6824	0.0007
Glutamic acid (μmol/L)	0.5143	0.02
Alanine (μmol/L)	0.5896	0.005
Arginine (μmol/L)	0.6998	0.0004
Phenylalanine (μmol/L)	0.5055	0.02
Glycine (μmol/L)	0.7351	0.0001
Methionine (μmol/L)	0.4297	NS
Ornithine (μmol/L)	0.6701	0.0009
Serine (μmol/L)	0.7165	0.0003
Tyrosine (μmol/L)	0.7545	<0.0001
Threonine (μmol/L)	0.8078	<0.0001
Tryptophan (μmol/L)	0.6359	0.002
Valine (μmol/L)	0.9299	<0.0001
Citrulline (μmol/L)	0.7900	0.0003
Histidine (μmol/L)	0.6461	0.007

NS = not significant. *p* < 0.05 (Spearman’s rho (ρ)).

## Data Availability

The data that support the findings of this study are available from the corresponding author upon reasonable request.

## Supplementary Materials

The following are available online at https://www.mdpi.com/article/10.3390/nu13082587/s1. Table S1: Nutrient concentrations in maternal circulation with natural conception and ICSI; Table S2: Nutrient concentrations in umbilical vein circulation with natural conception and ICSI; Table S3: Histopathology in placentae from naturally conceived and ICSI conceived pregnancies; Figure S1: ICSI does not alter mRNA expression levels of major placental transporter systems; Figure S2: Free amino acids in circulation of mothers who conceived naturally (CON) or with ICSI; Figure S3: Free fatty acid and lipids in circulation of mothers who conceived naturally (CON) or with ICSI.
