# Supplementary material for: Altered Umbilical Cord Blood Nutrient Levels, Placental Cell Turnover and Transporter Expression in Human Term Pregnancies Conceived by Intracytoplasmic Sperm Injection (ICSI)
Source: Nutrients. 2021 Jul 28;13(8):2587. doi: 10.3390/nu13082587 (PMC8399441; doi:10.3390/nu13082587)
Supplement: Supplementary file 1 [file nutrients-13-02587-s001.zip › nutrients-1260978-supplementary.pdf]

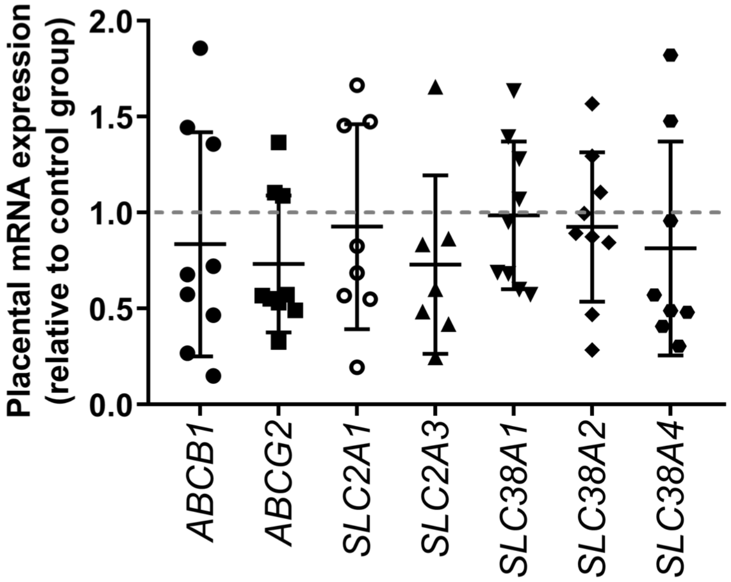

Supplementary Figure S1: ICSI does not alter mRNA expression levels of major placental transporter systems. Placental mRNA relative expression of the neutral amino acids transporters, *SLC38A1* (encoding SNAT1), *SLC38A2* (SNAT2) and *SLC38A4* (SNAT 4); of the glucose transporters, *SLC2A1* (GLUT1) and *SLC2A3* (GLUT3) and of the MDR efflux transporters, *ABCB1* (P-gp) and *ABCG2* (BCRP) in naturally conceived (n=10) compared to ICSI (n=9). Data are presented as media ± SD. Groups were analyzed using Unpaired T-test.

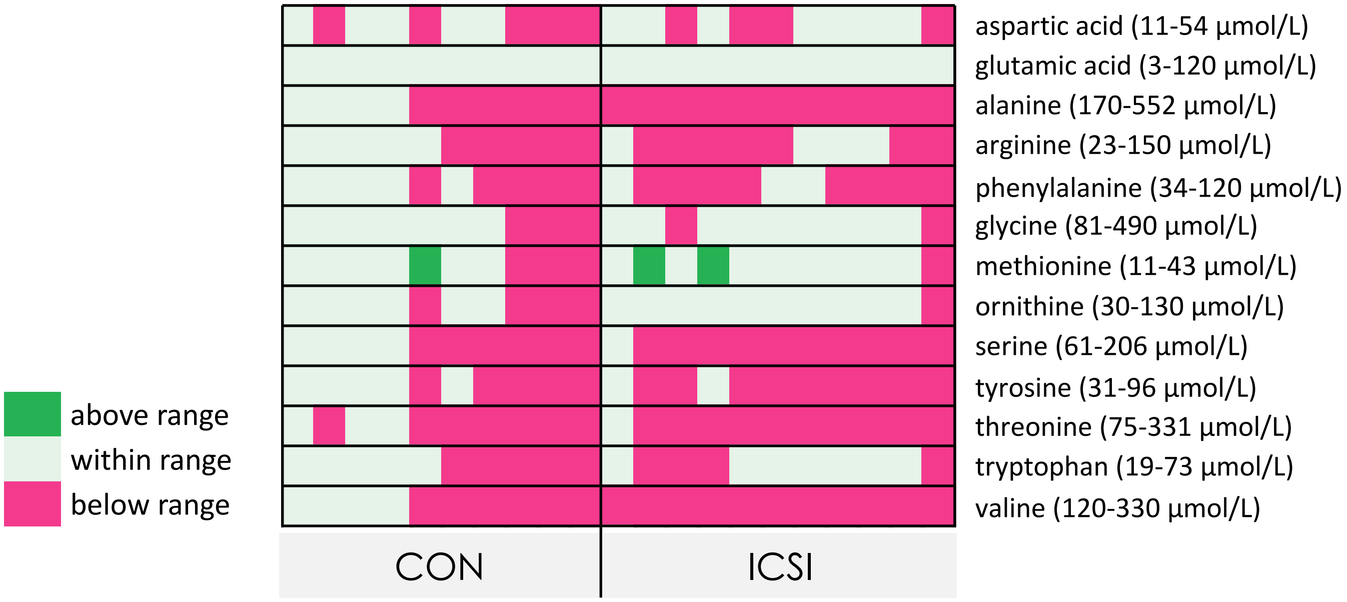

Supplementary Figure S2: Free amino acids in circulation of mothers who conceived naturally (CON) or with ICSI. Many mothers, irrespective of conception group, had levels of free amino acids below the reference range. There were more CON mothers with amino acid levels within the reference ranges than ICSI mothers. Data are amino acid levels categorized based on national reference data (see parentheses). Each column represents the nutrient profile of an individual pregnancy**.** Refer to supplementary tables S1 and S2 for values.

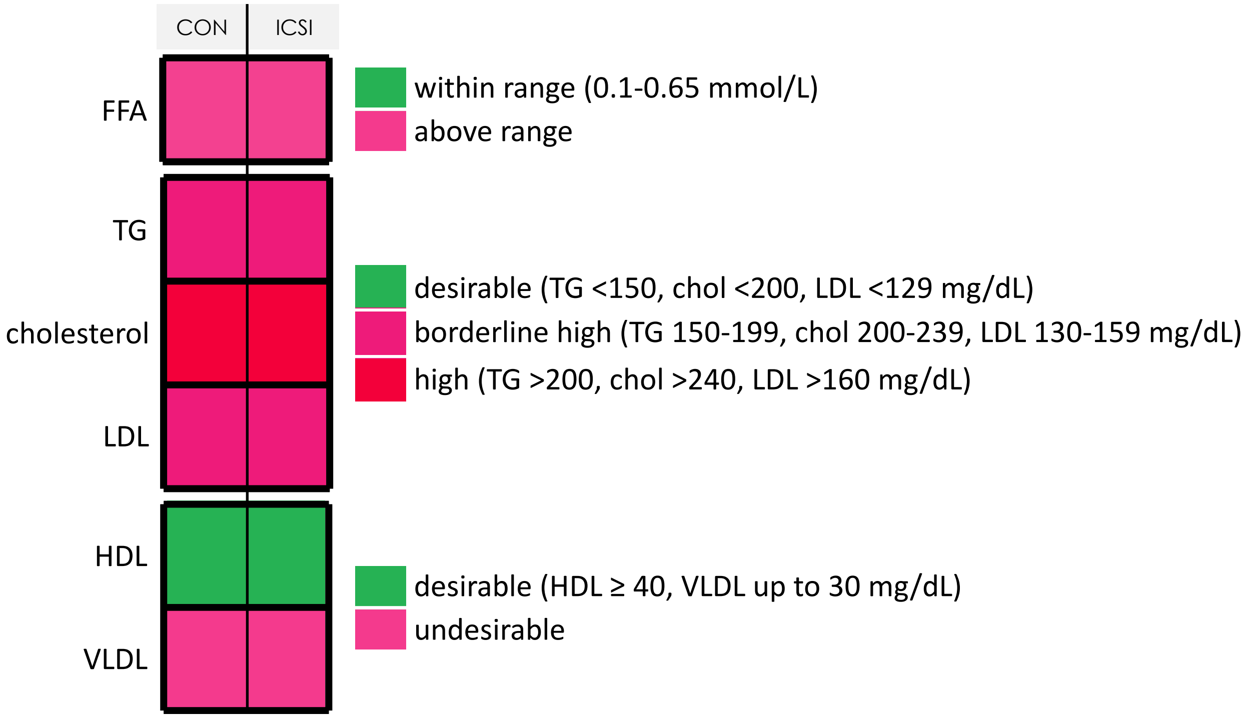

Supplementary Figure S3: Free fatty acid and lipids in circulation of mothers who conceived naturally (CON) or with ICSI. Many mothers, irrespective of conception group, had undesirable levels of lipid biomarkers. All mothers had desirable HDL levels. Data are mean biomarker levels and categorized based on national reference data (see parentheses). Refer to supplementary tables S1 and S2 for values. FFA = free fatty acids. TG = triglycerides. Chol = cholesterol. LDL = low density lipoprotein. HDL = high density lipoprotein. VLDL = very low density lipoprotein.

Supplementary Table S1: Nutrient concentrations in maternal circulation with natural conception and ICSI.

1 Nutrient	CON	ICSI	p-value
Glucose (mg/dL)	71.6 ± 6.9 (9)	71.2 ± 9.7 (11)	NS
Triglycerides (mg/dL)	174 ± 24.8 (9)	172 ± 66.2 (11)	NS
Cholesterol (mg/dL)	254 ± 82.3 (10)	251 ± 77.9 (11)	NS
HDL (mg/dL)	60.9 ± 15.8 (10)	57.6 ± 16.0 (10)	NS
LDL (mg/dL)	152 ± 61.5 (10)	156 ± 60.5 (11)	NS
VLDL (mg/dL)	34 ± 6.0 (9)	33.8 ± 12.9 (11)	NS
Aspartic acid (µmol/L)	11.5 (6.6-21) (10)	11.7 (9.2-13.6) (11)	NS
Glutamic acid (µmol/L)	62.4 ± 21.7 (10)	69.5 ± 12.5 (11)	NS
Alanine (µmol/L)	117 (84.9-289) (10)	97.3 (90.5-115) (10)	NS
Arginine (µmol/L)	19.1 (12.2-48.5) (10)	14.1 (10.6-22.9) (10)	NS
Phenylalanine (µmol/L)	33.5 (22.1-79.8) (10)	32.1 (30.5-33.4) (9)	NS
Glycine (µmol/L)	126 ± 61.2 (10)	103 ± 25.2 (11)	NS
Methionine (µmol/L)	15 (9.5-19.7) (9)	13 (11.5-16.0) (9)	NS
Ornithine (µmol/L)	51.3 (22.3-73.1) (10)	36.7 (32.0-39.6) (9)	NS
Serine (µmol/L)	39.5 (28.6-124) (10)	32.8 (27.7-38.6) (10)	NS
Tyrosine (µmol/L)	34.5 (15.4-61.3) (10)	27.3 (25.1-28.6) (9)	NS
Threonine (µmol/L)	53.6 (34.9-80.3) (10)	33.7 (31.3-43.1) (10)	NS
Tryptophan (µmol/L)	19.7 (11.3-59.2) (10)	19.7 (15.0-21.8) (10)	NS
Valine (µmol/L)	92.3 (36.3-193) (10)	67.6 (61.3-73.1) (9)	NS
Asparagine (µmol/L)	54.3 ± 19.2 (4)	57 (1)	–
Isoleucine (µmol/L)	61.9 ± 15.4 (5)	44 (1)	–
Leucine (µmol/L)	120 ± 10.3 (4)	118 (1)	–
Lysine (µmol/L)	119 (59.9-128) (5)	142 (142-142) (1)	–
Taurine (µmol/L)	86 ± 10.9 (4)	93 (1)	–
Histidine (µmol/L)	75.9 (26.8-182) (6)	67.4 (35.6-147) (10)	NS

Data are mean ± SD or median (IQR). n for each variable indicated in parentheses. NS=not significant. – = unable to compare groups due to low n. CON=control naturally conceived; ICSI=intracytoplasmic sperm injection.

Supplementary Table S2: Nutrient concentrations in umbilical vein circulation with natural conception and ICSI.

1

Nutrient	CON	ICSI	p-value
**Glucose (mg/dL)**	64.8 ± 13.1 (10)	63.9 ± 11.1 (9)	NS
**Triglycerides (mg/dL)**	30.1 ± 9.0 (10)	26.5 ± 12.1 (11)	NS
**Cholesterol (mg/dL)**	57.2 ± 15.7 (10)	52.2 ± 6.4 (9)	NS
**HDL (mg/dL)**	26.4 ± 4.9 (10)	24.5 ± 6.0 (11)	NS
**LDL (mg/dL)**	25.3 ± 11.4 (10)	23.6 ± 5.6 (10)	NS
**VLDL (mg/dL)**	6.6 ± 1.8 (10)	5.9 ± 2.2 (11)	NS
**Aspartic acid (µmol/L)**	12.3 (7.8-28.3) (10)	11.1 (10.5-14.6) (11)	NS
**Glutamic acid (µmol/L)**	84.7 (62.2-118) (10)	83.7 (65.8-103) (11)	NS
**Alanine (µmol/L)**	153 (110-321) (10)	124 (93.1-150) (10)	NS
**Arginine (µmol/L)**	23.0 (10.6-62.8) (10)	18.9 (12.9-22.6) (10)	NS
**Phenylalanine (µmol/L)**	41.2 (30.8-74.0) (10)	38.0 (36.6-41.8) (10)	NS
**Glycine (µmol/L)**	175 ± 63.6 (10)	147 ± 11.5 (9)	NS
**Methionine (µmol/L)**	15.8 ± 4.7 (9)	20.2 ± 8.3 (11)	NS
**Ornithine (µmol/L)**	62.6 ± 22.6 (10)	62.5 ± 18.0 (11)	NS
**Serine (µmol/L)**	51.1 (38.3-140) (10)	53.0 (37.1-60.3) (10)	NS
**Tyrosine (µmol/L)**	39.1 (26.6-81.8) (10)	36.3 (32.6-42.4) (11)	NS
**Threonine (µmol/L)**	60.3 (45.7-85.5) (10)	47.9 (43.2-58.9) (10)	NS
**Tryptophan (µmol/L)**	27.7 (18.7-51.8) (10)	26.4 (19.3-28.6) (10)	NS
**Valine (µmol/L)**	100 (66.2-151) (10)	82.2 (75.1-84.9) (9)	NS
**Asparagine (µmol/L)**	88.5 (47.3-94.5) (4)	88 (88-88) (1)	–
**Isoleucine (µmol/L)**	91.3 ± 12.9 (4)	71 (1)	–
**Leucine (µmol/L)**	145 ± 24.7 (4)	102 (1)	–
**Lysine (µmol/L)**	151 ± 40.1 (4)	174 (1)	–
**Taurine (µmol/L)**	121 ± 29.7 (4)	91.3 ± 53.3 (2)	NS
**Histidine (µmol/L)**	66.3 (30.4-159) (6)	66.3 (30.9-124) (1)	NS

Data are mean ± SD or median (IQR). n for each variable indicated in parentheses. NS = not significant. – = unable to compare groups due to low n. CON=naturally conceived; ICSI=intracytoplasmic sperm injection.

Supplementary Table S3: Histopathology in placentae from naturally conceived and ICSI-conceived pregnancies.

Placental Pathology	CON (n=10)	ICSI (n=11)	p-value
increased perivillous fibrin	3 (30%)	6 (54%)	NS
chorioangiosis	2 (20%)	2 (18%)	NS
vascular congestion	5 (50%)	6 (54%)	NS
villous trunk edema	6 (60%)	3 (27%)	NS
calcification	1 (10%)	1 (9%)	NS
perivillous hematoma	2 (20%)	0 (0%)	NS
infarction or ischemia	0 (0%)	2 (18%)	NS

Pathological features were deemed either present or absent in control naturally conceived (CON) and ICSI placentae. Data are n and proportion (%). $P<0.05$ (Fisher's exact test).
